# Neural Tube Defect Repair And Ventriculoperitoneal Shunting: Indications And Outcome

**Published:** 2012-04-01

**Authors:** Shandip K Sinha, Anjan Dhua, Mohit Kumar Mathur, Sudhir Singh, Manoj Modi, Simmi k Ratan

**Affiliations:** Department of Pediatric Surgery, Maulana Azad Medical College, New Delhi-110002; 1 Department of Neonatology, Maulana Azad Medical College, New Delhi-110002

**Keywords:** VP shunt, neonatal surgery, neural tube defect, meningomyelocele

## Abstract

Neural tube defect with its global involvement of nervous system has lot of implications. There is cotroversy in terms of timing of repair, simultaneous or metachronous ventriculoperitoneal shunt and criteria for shunt surgery in neonatal age. We are reporting our approach and results of management of this disease in neonatal period.

## INTRODUCTION

Neural tube defect (NTD) has global impact on central nervous system with hydrocephalus and Arnold-Chiari malformation (ACM) as one of the commonest associations. Management of hydrocephalus is controversial in terms of timing of repair, simultaneous repair with NTD and criteria utilized for shunting [1-3]. We want to present our short experience of the management of newborns of NTD with hydrocephalus and review the literature for controversies associated with it.

## MATERIALS AND METHODS

It is a retrospective case series in which all cases of NTD operated in neonatal period in Department of Pediatric Surgery of a tertiary care hospital from January 2010 to January 2012 were included. The type of defect, its location, associated neurological deficits, and clinical features of hydrocephalus were recorded.

The preoperative investigations done included X-ray spine and neurosonogram (lateral ventricle diameter, V: H ratio). Ultrasound abdomen and echocardiography were done to evaluate associated anomalies.

Preoperative MRI spine was done in selected neonates whose clinical examination or neurosonogram suggested spinal malformations (severe kyphosis and/or scoliosis, hemi-myelocele, split cord malformation etc.). If indicated CSF-tap for protein, sugar and cells with culture, was also done.

The neonates needing simultaneous shunt and neural tube repair were taken for operation under general anaesthesia. Standard techniques were followed for shunting following which patients were repositioned to prone position. Repair of the neural tube was then done.

All children received antibiotics during peri-operative period. Children were followed up with head circumference and neurosonogram fortnightly.

## RESULTS

Eight cases of neural tube defects were operated during study period. The distribution of neural tube defect was as follows: lumbosacral-5, lumbar-1, and thoracic-2 (Figure 1). Male:female distribution was 5:3. The median age and weight at operation were 5 days and 2.48 kg, respectively. In five cases, diagnosis was made on antenatal USG scans. Six patients were born through vaginal route, whereas two had caesarian section because of obstetrics reasons.

**Figure:**
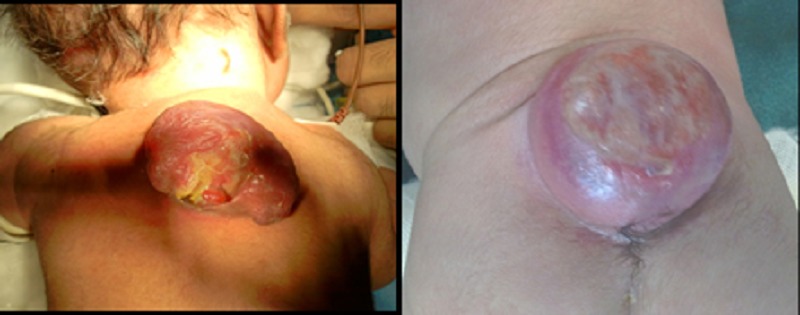
Figure 1: Thoracic meningocele and Lumbosacral MMC in newborns.

Four patients had meningomyelocele (MMC), whereas four had meningocele. Two of the patients had cerebrospinal fluid leak at time of delivery and needed urgent surgery. Of the patients with meningocele, all had normal lower limb muscle power with no clinically detectable bowel/bladder involvement. Of the patients with MMC, two had decreased anal tone with no limb dysfunction, and two had lower limb weakness with bowel/bladder involvement. None of the patients had associated anomalies.

Two neonates had hydrocephalus at birth and needed simultaneous VP shunt (Table 1). USG criteria utilized to identify patients needing simultaneous VP shunt was dilated lateral ventricle (>15mm). There were no anaesthetic or post operative complications and all had uneventful postoperative outcome except for one case that had prolonged postoperative ileus and managed conservatively. Close follow-up showed development of hydrocephalus needing VP shunt in one patient within 1 year, whereas one patient died of pneumonia at 3 months of age.

**Figure:**
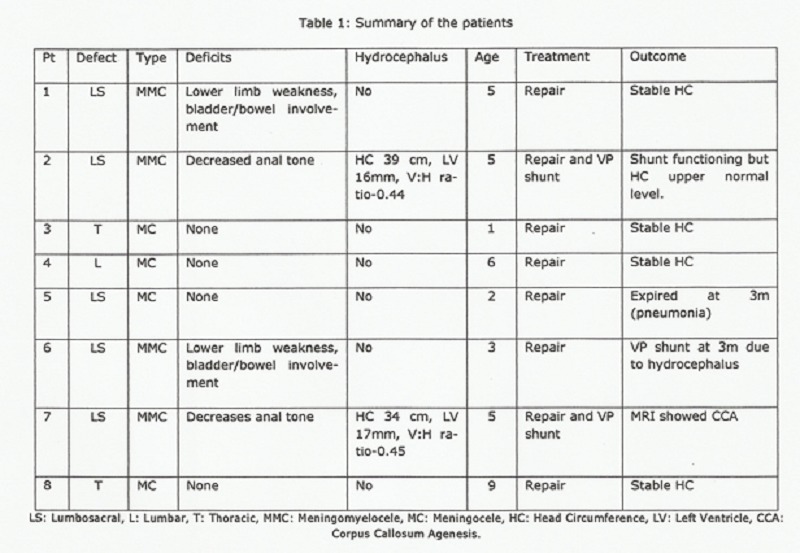
Table 1: Summary of the patients.

## DISCUSSION

Neural tube defects and hydrocephalus are closely associated. Hydrocephalus can be present at birth, or can develop after repair of MMC. Whether the shunt operation is to be done simultaneously with NTD repair or in a staggered manner is controversial and there are opponents and proponents of either philosophy.

The head circumference is often used as criteria for follow up of the patients for development of hydrocephalus, but its normal value in newborn cannot rule out hydrocephalus [1]. The criteria used for diagnosis of hydrocephalus are described in Table 2 [2]. In our series, one newborn had ventriculomegaly but normal head circumference. Clinical examination of the myelodysplastic neonate usually does not reveal evidence of hydrocephalus and ventriculomegaly on ultrasonography predicts the later development of hydrocephalus following meningomyelocele closure [1]. It is recommended that neuro-imaging (USG or CT skull) should always be done in newborns for confirming the diagnosis of hydrocephalus or getting the basal levels of ventricle diameters for follow up. The growth rate of a head circumference and growth of Evans' index predicts a progressing hydrocephalus during the first few weeks [3].

**Figure:**
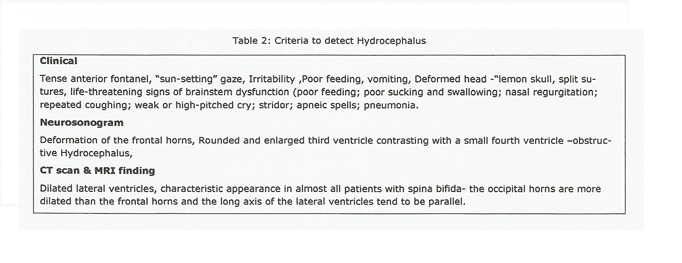
Table 2: Criteria to detect Hydrocephalus

The timing of repair of neural tube defect is controversial with recommendations of immediate repair in newborn period to delayed repair [4]. The advantages of repair in newborn period include preventing CSF infection and further neurological deterioration. Moreover, primary neurosurgical repair of MMC within the first 72 hours after delivery provides an improved neurogenic bladder/bowel prognosis compared to repair at a later time [5]. In fact an increased risk of shunt malfunction has been reported following delayed MMC repair because of increased CSF proteins and debris, which may lead to shunt occlusion even without infection [6]. Our approach aims to repair the open neural tube de¬fect early (within 72h). However, only three of our pa-tients were operated within this time period because of delayed referral. Negative CSF culture was always ensured.

Hydrocephalus is frequently associated with MMC (85-90%) that it may be considered part of the malformation. In less than 15% of the cases, hydrocephalus is already overt at birth, manifesting with the classical signs of raised intracranial pressure (ICP) (split sutures, tense anterior fontanel, sunsetting eyes, vomiting, etc.), or even with the life-threatening signs of brainstem dysfunction (poor feeding, poor sucking and swallowing, nasal regurgitation, repeated coughing, weak or high pitched cry, stridor, apneic spells, pneumonia etc.), secondary to the impaction of neural structures within the small posterior fossa (due to the Chiari II malformation) [7,8]. This particular subset of myelodysplastic newborns with significant hydrocephalus warrants early surgical treatment for hydrocephalus. Two of our cases needed early VP shunt based on clinical and USG criteria.

The neonates who have requirement of VP shunt can be managed by either early MMC repair followed by VP shunt (after 2-3 weeks), or simultaneous VP shunt and MMC repair [9,10]. The selection criteria for simultaneous shunt were lateral ventricle >15mm and V:H ra¬tio>0.4. Advantages of simultaneous repair approach include administering only one anesthetic, diminution in incidence of cerebrospinal fluid leaks from the repair, protecting the brain from the deleterious effects of progressive ventricular dilatation, shortened hospital stay and the resultant cost-effectiveness [11,12]. Disadvantages include increased infection of shunt because of reversed CSF flow from lumbar region to ventricles, and need of surgery in newborn patients who may also have a weakened immune system [13-15]. We have followed the simultaneous approach of VP shunt and neural tube defect repair with good results with no infective complications.

To conclude, neonatal VP shunt surgery is indicated in select cases of neural tube defect. It can be performed with minimal morbidity although risk of infection and meningitis are there.

## Footnotes

**Source of Support:** Nil

**Conflict of Interest:** None declared

**Editorial Comments**

VP shunts in neonates are known to have a higher complication rate. The alternative use of acetazolamide, at a starting dose of 25mg/Kg/day which can be escalated up to 100 mg/Kg/day under supervision, when the V:H ratio is >0.4 on neurosonogram; is found to be safe in neonates and children. However, one should watch for the occasional occurrence of metabolic acidosis as a potential side effect. Editor – Journal of Neonatal Surgery

